# Spontaneous posterior rectus sheath hernia: A case report and literature review

**DOI:** 10.1016/j.ijscr.2022.107834

**Published:** 2022-12-13

**Authors:** Fatima Alshayeb, Layth Al-Karaja, Ahmadullah Musleh Abu Yousef, Jalal Ahmad Taqatqa, Rafiq M.A. Salhabb, Mohammad Eid Al Mohtasib

**Affiliations:** a Faculty of Medicine, Jordan University of Science and Technology, Jordan; b Faculty of Medicine, Al-Quds university, Jerusalem, Palestine; c General surgery Department, Ahli hospital, Hebron, Palestine; d Faculty of Medicine, Palestine polytechnic university, Palestine

**Keywords:** Rectus sheath hernia, Hernia, Rectus sheath, Abdominal wall, Case report

## Abstract

**Introduction:**

Hernias of the posterior rectus sheath are very rare abdominal wall hernias with only around 15 reported cases to date.

**Clinical presentation:**

This case report examines a 27-year-old female who is presented with epigastric abdominal pain and vomiting. An Abdomen CT scan was done and showed signs of SBO and herniation of the small bowel at the posterior rectus sheath. The patient underwent exploratory laparotomy that showed right-sided posterior rectus sheath obstructed hernia, which was repaired with primary closure. Postoperatively, the patient was doing well and was discharged on postoperative day 3 in good general condition.

**Conclusion:**

The patient had no complaints during her follow-up at one month. Due to its rarity and potential complications, it is also important to report this case to enhance the evidence base for posterior rectus sheath hernia and to familiarise this uncommon condition to radiologists, clinicians, and surgeons.

## Highlights

• Development of posterior rectus sheath hernia is quite rare condition that has been reported only 13 times in the literature.
• Posterior rectus sheath hernia should be considered in differential diagnosis of abdominal pain.
• Multiple treatment modalities have been described for this condition; surgery is still the treatment of choice.
• Surgical repair has been used in this patient.

## 1 Introduction

A hernia is known as any abnormal protrusion through the wall of the cavity of any organ or tissue. They can be present as either congenital or acquired. Hernias may have several locations with the most common locations being inguinal, incisional, femoral, or ventral [1]. One of the least common hernias lies within the muscles of the abdominal wall but does not cross the subcutaneous tissue is called the interparietal hernia [2]. A posterior rectus sheath hernia is a subtype of the interparietal hernia group [3]. It was found that rectus sheath hernias result from trauma or any surgical weakening of the wall. It has only been documented twelve times from 1937 to 2019.

This work has been reported in line with the SCARE criteria, which is used by authors, journal editors, and reviewers to increase the robustness and transparency in reporting surgical cases [14].

## 2 Case presentation

A 27-year-old female patient was presented to the emergency room complaining of acute abdominal pain. The pain was mainly in the epigastric region with acute onset, that increased gradually in intensity, radiated to the back, and associated with one episode of vomiting of gastric contents. The patient had no past history of fever or constipation and didn't report history of any abdominal mass in the past or previous similar episode. The patient had 2 previous cesarean sections. The patient had no known drug allergy with a non-contributory family history.

On physical examination, vital signs were stable with which her blood pressure was 127/91 mmHg and pulse rate was 84 beats per minute (bpm), Spo2 95 %, Glasgow coma scale was 15. The patient looked in pain, the positive findings in the abdominal examination were well healed Cesarean section scar, epigastric and infra-umbilical tenderness with no palpable swelling or overlying skin changes, no warmth, no erythema, and all inguinal orifices were intact.

No other findings on complete physical examination.

Laboratory findings were as follows:

Complete blood count results: WBC 7.16 × 10^3, RBC 5.13 × 10^6uL, Hb% 13.49, HCT 44.5 %, MCV 86.6 x um^3, MCH 26.2 PG/ML, MCHC 30.3 %, PLT 206.5 × 10^3/ul, Neutrophils 79.4 %, Lymph 16.7 %, Monocytes 3.08 %, Eosinophils 0.15 %, Basophils 0.59 %, RDW 14.43.

Chemistry results: BUN 11 mg/dL, Creatinine 0.75 mg/dL, Random blood sugar 120, SGPT 16 uU/mL, SGOT 17 U/L, Total bilirubin 0.5 mg/dL, Alkaline phosphatase 62 U/L, Lipase 33 U/L.

Electrolytes results: Na + 137 mEq/L, K+ 4.1 mEq/L, Chloride 104 mmol/L.

Computed tomography (CT) of his abdomen and pelvis was performed and demonstrated splitting of the posterior sheath of the right-sided rectus abdominus muscle and showed bowel herniation through a defect within the sheath which caused small bowel loops dilation proximal to the entry point that was measured up to 4 cm in diameter and relatively collapsed bowel loops distally, represented posterior sheath hernia that was complicated by intestinal obstruction.

**Fig. 1:**
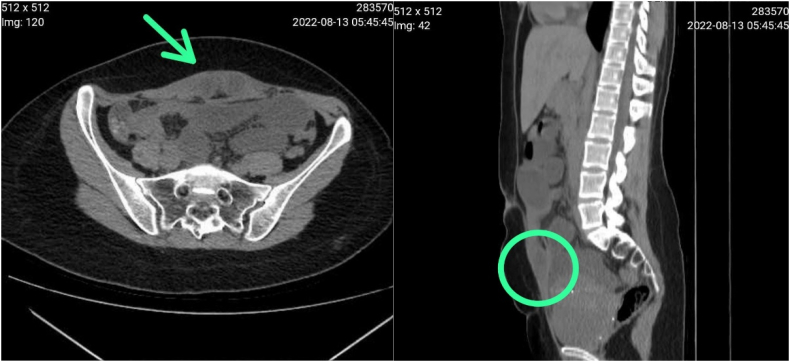
Computed tomography scan showing posterior rectus sheath hernia.

Mild free pelvic fluid, the intrauterine device was also noted, with the normal-appearing of the liver and pancreas. Fig. 1.

**Fig. 2:**
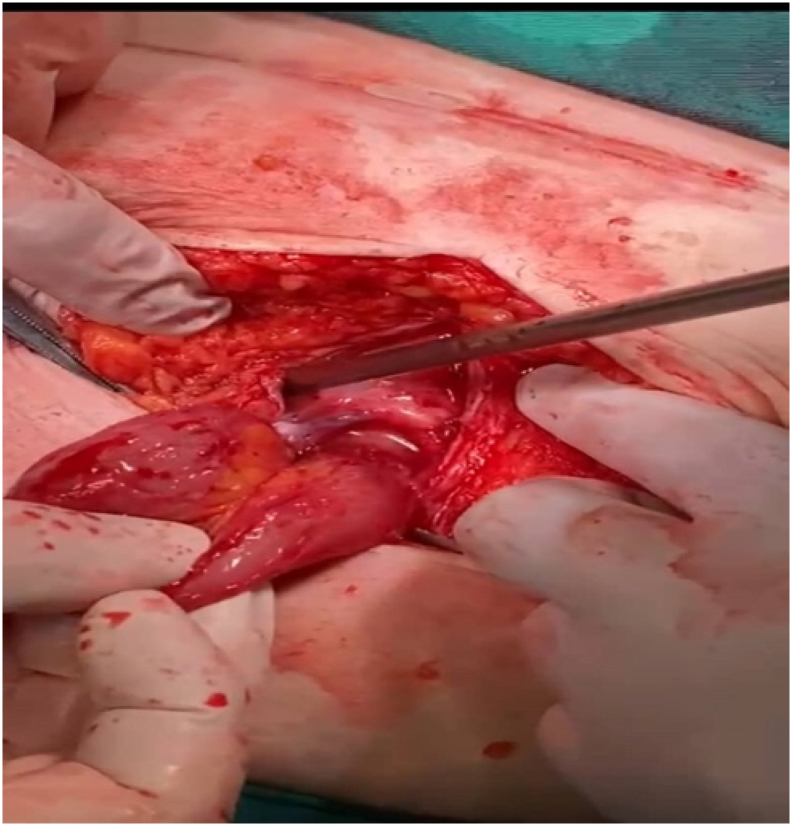
Intraoperative section of the hernia.

Obstructed posterior rectus sheath hernia as the final diagnosis was made and the surgical repair was the decision. The operative procedure was made by a lower midline incision, opened layer by layer, then muscle was splitted and the peritoneum opened. The findings were abdominal wall edema, infra-umbilical obstructed hernia with small bowel content (loop of terminal ileum) “Fig. 2” in the abdominal wall between fascia and rectus muscle with severe adhesion, there were two pockets dirty in the abdominal wall where the hernia obstructed, formal exploration of the small bowel was done till the ileocecal valve which was found normal, no other pathology was identified. Adhesiolysis, irrigation, and cleaning of the two pockets many times, and irrigation and cleaning of the abdominal cavity were done. Closed by mass closure in which we used continuous Polydioxanone (PDS) 1 suture.

Hemostasis secured, count correct, closure of the fascia, and closure of the skin where the end of the surgical intervention. [Fig. 2].

## 3 Discussion

Exceedingly rare, with only 15 reported cases, the type of hernia is the posterior rectus sheath hernia. The first case documented was in 1937. Later on, in 2009 there were only a total of eight cases reported with an additional three cases that were in 2014, 2017, and 2018 [4], [5], [6], [7].

According to the literature, posterior rectus sheath hernias were more common in females than males, and the age of diagnosis ranged widely between 25 and 83 years of age [1], [7], [13]. Also, hernias are reported as being 2 cm approximately in size and the largest size they reached was 6 cm [5]. Patients presenting with hernia have many clinical presentations that may be asymptomatic or symptomatic like nausea, vomiting, and abdominal pain [2], [3], [6], [7].

Inguinal hernias are hernial sacs that lie between abdominal muscle layers. Rectus sheath hernias, a rare type of inguinal hernias, are considered intraparietal hernias. There are 3 subtypes of intraparietal hernias (peritoneal, interstitial, and superficial) with the most common type being interstitial. In our patient, the hernia was preperitoneal as it was located between the transversalis fascia and the peritoneum.

The exact pathophysiology of this rare type of hernia is not fully understood to this date [2], [7]. In abdominal wall anatomy, the rectus sheath, also known as the rectus fascia, is formed by the transverse abdominus muscle, internal and external oblique muscles as well as the pyramidal and rectus abdominis muscles. These muscles prevent spontaneous herniation due to their strong resistance. The rectus sheath can be anatomically divided into anterior and posterior laminae with the anterior rectus sheath being considered stronger in comparison as the posterior rectus sheath is only comprised of the transverse abdominis muscle [7], [9].

One of the possible explanations for weaker structures being invaded by internal structures is that the Linea Alba of the posterior sheath component is thinner than the anterior sheath component [10]. Similar to other hernial types, it has been hypothesized that conditions that can increase thoracoabdominal pressure, like obesity, ascites, and pregnancy, can increase the incidence of such cases [3].

Rectus sheath hernias are usually diagnosed with imaging studies like CT scans, Ultrasound, and MRI. The best diagnostic modality is a CT scan due to its high sensitivity to pinpoint the complications inside the hernia sac such as incarceration, obstruction, and strangulation [1 + Fig. 1]. Although laparoscopy has been used for diagnostic and therapeutic purposes, a definite diagnosis is made during surgery [3], [13]. In larger defects with prosthetic repair, the treatment of choice preferred is primary closure. Our case was diagnosed with the use of a CT scan, the patient then underwent surgery and intraoperatively we found the cause of the symptoms was due to the presence of bowel obstruction.

Rectus sheath hernias, especially posterior type, can be either found as being congenital in origin or could be post-surgical. There were cases in which patients were treated conservatively then discharged and then later on throughout the years they had exploratory laparotomy for hernia repair [5]. Conservative treatment includes serial examination check-ups with vital signs in the presence of observation and follow-up.

## 4 Conclusions

In this case, spontaneous posterior rectus sheath hernia was a diagnosis with a significant clinical course and was thus managed surgically. This type of hernia is an uncommon entity and they may be asymptomatic or present with severe abdominal pain and incarceration, which can progress to strangulation and ischemia with an increased risk of bowel gangrene if left untreated. Prompt diagnosis of this hernia with or without surgical intervention is needed for a successful outcome. Our case report adds to the limited stock of available literature on this unusual issue and strengthens the evidence base on the best approach to support informed clinical decision-making in this rare type of hernia. The significant clinical implication of reporting such cases is the increased identification rate of rare clinical conditions which otherwise often go unnoticed leading to avoidable complications.

## Ethical approval

We obtained written informed consent from the patient for reporting this case and accompanying images. A copy of the written consent is available for review by the Editor-in-Chief of this journal on request.

## Funding

The study did not receive any funding.

## Author contribution

Study concept or design: Fatima Alshayeb, Layth Al-Karaja

Writing the manuscript: Fatima Alshayeb, Layth Al-Karaja, Ahmadullah Musleh Abu Yousef, Jalal Ahmad Taqatqa

Review & editing the manuscript: Fatima Alshayeb, Layth Al-Karaja, Mohammad Eid Al mohtasib

Data collectors: Jalal Ahmad Taqatqa, Ahmadullah Musleh Abu Yousef.

## Guarantor

Dr. Mohammad Eid Almohtasib

## Research registry number

The study has no trial registry number.

## Provenance and peer review

Not commissioned, externally peer-reviewed.

## Registration of research studies

Not applicable.

## Declaration of competing interest

There is no conflict of interest to declare.
